# Research to Help Projects Operate Successfully

**DOI:** 10.9745/GHSP-D-23-00433

**Published:** 2023-12-18

**Authors:** Robert Hornik

**Affiliations:** a Annenberg School of Communication, University of Pennsylvania, Philadelphia, PA, USA.

## Abstract

Operational research is central to project success; an explicit conceptual and operational model can underpin questions that merit attention, prioritizing those where doubt is high and where responsive changes are likely.

## Key Messages

- Operational research is not only about data collection but also about knowing what questions are worth addressing and how to incorporate results into program decisions in a timely way.
- There is likely to be value in specifying program conceptual and logistical models that will lay out possible foci for operational research and generate potential lists of activities to monitor and expectations for paths of effect that can be tested.
- Research should be prioritized where doubt about component success is higher and projects are open to modification while balancing rigor of research designs with the utility of results.

## INTRODUCTION

Most of my work has involved large-scale communication interventions: national or regional campaigns to influence behavior including, as examples, child survival campaigns to encourage vaccination or use of oral rehydration solution, anti-drug and anti-tobacco campaigns to encourage reduced use among adolescents, and campaigns to encourage screening for or prevention of specific cancers among adults. Such large-scale communication programs must often operate at a physical distance from their audiences. In-person programs may have some possibility of assessing the immediate responses of audiences. In contrast, mediated programs will lack direct, immediate responses and likely have a special need for structured feedback systems to make up for that lack. From that perspective, I write about the potential value of research meant to shape ongoing and evolving operations. While my experience has focused on large-scale mediated interventions, I expect the concerns described here can be generalized to other interventions at scale. Parts of this article also parallel similar arguments expressed during the early period of the AIDS epidemic.^1^ Similar arguments have been made throughout this supplement and elsewhere.^2^^–^^4^

Campaigns often have, at their initiation, used careful foundational research to establish their broad approach and formative research to choose ideal messages. After they have operated for a while, they may have been subject to intensive summative evaluations of their success. In contrast, I would argue that such large-scale communication campaigns often have underinvested in the sort of operational research that is likely to maximize their effectiveness. There has been much less research that collects and uses regular and systematic feedback to help programs operate more effectively than there has been early foundational, formative, and late summative evaluation. In this commentary, I provide a few examples of programs whose failure to use such evidence in a timely way meant they may have missed the opportunity for greater success. Those shortfalls may reflect either that they did not gather relevant tracking information or, more importantly, that they could not analyze and did not use that information for readjusting their operations in a timely way.

Then, I will discuss what a useful operational research function might look like—not only making an argument for gathering data but also advocating for a process of defining what questions should be a priority for operational research and how programs can be ready to make use of collected data to correct operational weaknesses. I will argue for the explicit development of both conceptual models (the paths through which a program is expected to influence behavior) and logistical models (laying out the linked concrete activities that the program is expected to realize). These models detail how a program is expected to produce effects. They will be the basis for judgments about where the biggest concerns about failure may lie and, thus, which program components merit focus for ongoing operational research.

Conceptual and logistical models detail how a program is expected to produce effect and will be the basis for judgments on where failure may lie and which program components merit focus for ongoing operational researtch.

## CASE STUDIES

In the examples that follow, I examine 2 cases where operational data were being collected but where that data may not have been fully used. In elaborating on these cases, I want to emphasize that I think the problem of operational research may often lie not in collecting data but in collecting data that respond to questions that are relevant to operational questions and having a management structure that will use available data to make new operational decisions.

### Case 1

The U.S. National High Blood Pressure Education Program was an essential and early model of an effort to improve public health. It involved public education (media campaigns), medical advocacy (through outreach to clinical societies), and other components to raise public awareness of the risks of high blood pressure and motivation to have it treated. By some estimates, it was highly successful, accelerating the decline in stroke mortality quite substantially. Its evaluators tell an intriguing story about its evolution. They gathered national sample data every 3 years between 1971 and 1991 and noted that over that period, there were sharp increases in awareness of hypertension and in the proportion of people with hypertension with their blood pressure under control (as well as increases in visits to physicians and decreases in salt consumption).^5^ Of particular interest, they noted that between the first 2 data collection periods 3 years apart (1971–1972 vs. 1974–1975), there was a large increase in awareness of hypertension (from 50% to 64%) but a much smaller increase in the proportion of people with hypertension who had their hypertension under control (17% to 20%). The program managers pointed to this finding as the basis for a decision to shift strategies from an emphasis on stimulating awareness toward a strategy focused on “long term therapy, maintenance and control of high blood pressure.”^6^ This shift forecasted large subsequent changes in the proportion of hypertensives who had their blood pressure under control (16% in 1972 to 55% by 1991). This claim is important in 2 ways. First, it testifies to the importance of being open to change and the need to respond to evidence about ongoing effects. Second, in contrast, it suggests some concern about the slow pace of program evolution. If the monitoring information had been collected more often than the 3-year gap illustrated here, it might have accelerated the evolution of the program from its focus on building awareness to its emphasis on action.

### Case 2

There has been extraordinary success in improving child survival. In Ethiopia, for example, child mortality declined from 205 deaths per 100 live births in 1990 to 64 in 2013. Among the recommended programs for supporting the continuing decline in child mortality is retraining and supervising health post workers so that they can effectively deal with prevention and treatment consistent with the Integrated Community Case Management of Childhood Illnesses (ICCM) model. A careful evaluation of an “at scale” 2011–2013 implementation of ICCM was conducted in Ethiopia.^7^ This program retrained and supervised health post workers in the appropriate treatment of childhood illnesses. The cluster-randomized evaluation compared geographic areas that received this intervention with areas that operated under the conventional health workers' system. Both sets of areas made progress in treatment demand and in mortality but only at the same rate; there was no advantage to the ICCM areas. The evaluators concluded that the failure of the program was due to its inability to stimulate demand; parents were not bringing children at risk into the health posts, so retrained health post workers did not have the opportunity to make use of their newly learned skills. The program only improved supply quality and did not affect demand. Yet the program's developers knew about this early on. The measured demand for health post services was 4% at baseline; a monitoring study 6 months into the program's operation had already found no increase in demand.^8^ The program was (apparently) unresponsive to this evidence: no demand creation component was added. We don't know why this did not affect the evolution of the program. Perhaps this reflected the delay in processing the midterm evidence, or perhaps it reflected the commitment to undertaking an unmodified evaluation of the intervention as originally proposed. So, in using this as an example, perhaps unfairly, I draw on it as if it were an operational program. It testifies in that context for a need to build capacity to capture evidence about program functioning as it operates and, in parallel, to develop institutional capacity to respond to that evidence and to reshape the program.

From each of these cases, we draw an idea. The National High Blood Pressure Education Program was responsive to evidence, but the evidence was gathered on a slow cycle, delaying redirection. The Ethiopia ICCM had evidence available, but that evidence was not used to redirect its operating model. These examples underpin the next section of this article—the capacities that should be part of a monitoring system meant to help projects work.

## OPERATION RESEARCH SYSTEM CAPACITIES

We often think about feedback systems in terms of the data collection procedures, perhaps thinking about the potential for new technology to make such data collection richer and quicker. The ability to collect data in a timely and cost-effective way does matter, of course. However, I argue that the capacity for systematic and timely data collection must be complemented with 2 other capacities: the capacity to decide what needs to be assessed as it evolves over time and the openness to respond to collected data, to make new decisions contingent on what the evidence says. These capacities may be rarer than the ability to collect data.

The capacity for systematic and timely data collection must be complemented by the capacity to decide what needs to be assessed as it evolves over time and the openness to respond to collected data.

How can a program decide what operational research questions are worth addressing? The basic approach endorsed here involves 2 steps: (1) the development of an operational model of how the program is expected to work that will produce a long list of potential issues addressable in operational research and (2) the development of a logic for prioritizing among that long list of questions for allocation of research resources. As an example of such an operational model, the Figure describes major aspects of the U.S. National Youth Anti-Drug Media Campaign (NYAMC) when it was developed in the late 1990s.^9^ This project eventually spent more than US$1 billion to influence uptake of drugs. To explain how to use this model, we need to provide some explanation of its logic. Each of the boxes to the left of the center box (“exposure…”) captures an expected activity to be influenced by the NYAMC (e.g., “availability of campaign ads on mass media”). Each of the arrows to the right of the center box captures expected cognitive changes in the population as the result of the campaign (e.g., “overall attitudes toward drug use”). The arrows represent causal hypotheses. On the left side, if campaign ads are available, it is expected to lead both to direct exposure to messages and parent-child talk about drugs. On the right side, exposure to messages will lead to knowledge about the consequences of drug use and self-efficacy to avoid drug use.

All these boxes and arrows represent possible questions for operational research to address. Some represent monitoring opportunities, such as do parents (increasingly) talk with their children about drugs? Do communities (increasingly) organize anti-drug activity? Others (the arrows) represent operational hypotheses open to testing, such as does exposure to campaign messages lead to greater knowledge about consequences of drug use and affect perception of others' expectations for respondent's drug use?

Monitoring activities to make sure they happen in accordance with expectations can be a central part of operational research. Such monitoring can assure managers that events are happening as expected, or, if the activities are falling short of expectations, tell them there is a problem needing addressing. Monitoring may not permit confident attribution of observed changes to the campaign's influence; there might be other forces influencing changes (e.g., extraneous media coverage might influence parents' talk about drugs). However, evidence from monitoring will provide useful indicators of whether the model is operating as expected or not.

### Research Designs

Investigating the arrows in the model and establishing what campaign components are responsible for changes in expected outcomes will be a more demanding set of tasks. They will likely require a range of research designs and analytic approaches. The promise of particular research approaches will reflect the particular substantive context and available resources. As a brief illustration, consider the model-hypothesized mediated link between exposure to drug messages with friends' discussion of drugs, which, in turn, is expected to produce communication of anti-drug messages by friends. One can imagine a range of research designs to investigate whether this mediated link is operating as expected. Here are 2 (of many possible) contrasting examples.

- A simple observational approach would survey a sample of teens, ask them about their own exposure to ads, whether they have had discussions about the ads with their peer networks, whether they communicated drug-related messages to their peers, and whether the valence of those discussions was consistently anti-drug. If the association between own exposure and amount of discussion with peers was positive and valence of discussions was anti-drug, there would be some (albeit uncertain) evidence consistent with the hypothesized relationship.
- A more elaborate experimental approach might enroll a sample of social networks and randomly assign higher and lower doses of social media exposure to ads to networks and measure the amount and valence of drug discussion among those networks with expected results contingent on assigned treatment.

The first design is likely low cost and might be substantially representative of the population but would be weak inferentially. The second design would permit more confident inferences of causal effect but would be higher cost and perhaps less ecologically matched to the real circumstances of program operations. Evaluators would consider how crucial this part of the operating model to this linkage was, whether the results of either study would lead to change in project actions, and what the tradeoff was of spending scarce evaluation resources on one or another of these designs, relative to the value of addressing other questions with more and less expensive designs. This would lead them to choose one of these designs (or another) or choose to focus resources elsewhere.

**FIGURE:**
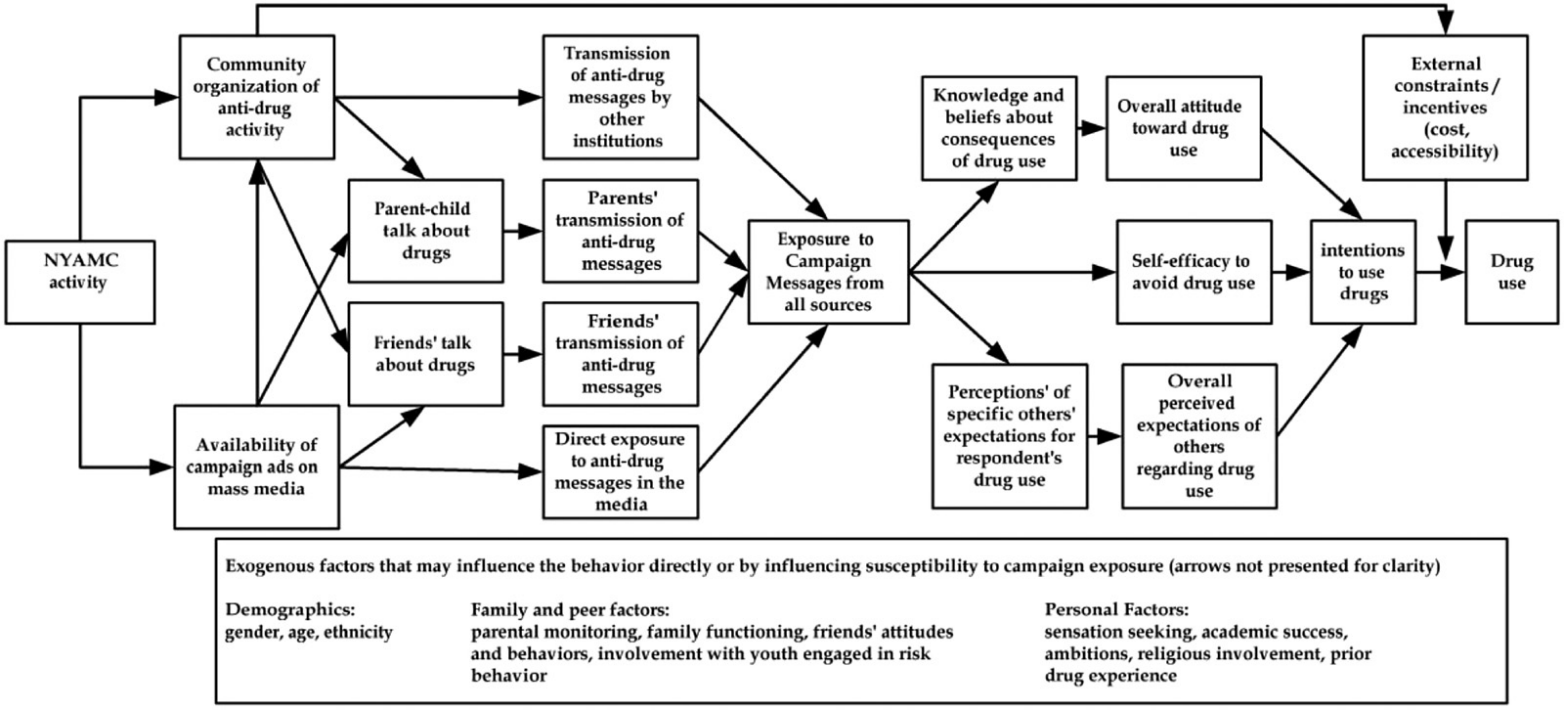
Combined Conceptual and Operational Model of the National Youth Anti-Drug Media Campaign Source: Hornik and Yanovitzky.^9^

## WHICH QUESTIONS TO ANSWER?

Even in this simplified model of effect (recognizing that every box could be subdivided, and every arrow could be elaborated with additional mediating variables), there are a very large number of monitoring and causal questions that might be addressed. No operational research program can possibly address them all. Once the model of the program is elaborated, what criteria can one use to choose among the potential questions? In the next section, we elaborate on a set of considerations that might underpin prioritization.

The suggested considerations include: What are the crucial links that must operate if a program is to be successful? Which answers will lead to meaningful changes in the operational model? What resources are there to get good enough answers to underpin decisions to change? Surely there will be complex additional considerations, including those represented by outside interests (e.g., donors and external political actors). Here, the focus is on those issues within the control of program managers.

What are the crucial links that must operate if a program is to be successful? Which answers will lead to meaningful changes in the operational model?

### Crucial Links

This consideration relies on 2 types of knowledge: substantive expertise in the behavioral area and intervention expertise in the type of intervention. Subject matter experts need to weigh in on what are the crucial influences on adolescent drug use? For example, which is most likely to be influential: parental expectations, peer expectations, or personal knowledge of risks of drug use? The project outlined in the Figure says that it will try to address all 3 paths of influence. Yet, operational evaluators may need to prioritize among these paths for their research investments. Which paths merit only monitoring? Do others require causal attribution studies, although some may merit lower investments permitting weaker inferences, while others call for more investment to underpin possible major modifications in the program's operating model? For example, if subject matter experts suggest that peer support for (or opposition to) the influence of direct ad exposure is crucial for drug use initiation, monitoring and testing the peer-relevant links would get priority. Other paths might require less attention.

Subject matter expert advice will help prioritize research questions. Also, expertise in the type of interventions being undertaken will be helpful. Those experienced in communication interventions will argue, for example, that many communication projects fail because of their failure to achieve broad reach and repeated exposure to their messages. That would suggest some priority on monitoring exposure to messages, making sure the exposure strategy is successful in reaching the expected proportion of the population and with expected frequency.

### Potential Influence on Decisions

The next consideration for choosing questions and deciding how much scarce resources to spend on addressing them is whether the answers will lead to changes in operations. Before data collection, program managers need to decide how the data will be used. Even priority questions will not merit substantial investment if there is little likelihood that possible answers will produce change in the operating model. Research planners might ask managers what they would change if the results of a study came out one way or another. Sometimes, program managers or other decision-makers may have no openness to change for particular program components. Such disinterest in change may reflect an a priori commitment to components of the current operating model. For example, the decision to include efforts to stimulate institutional actions in support of the operating model may reflect political considerations (e.g., in the Figure, the involvement of community organizations); evidence that the institutional path does not lead to additional exposure may not lead to reduced work with institutions. This disinterest in change might lead research planners away from studies of the role of institutions.

Also, even when research results might influence decisions, how much to invest in a particular question will consider how much investment is enough. The amount of investment can affect the precision of answers achieved, and sometimes, buying too much precision is not useful. For example, the program planners' operational model might seek to achieve 75% exposure to the primary messages. However, small variations from that goal might not affect media buying plans. If monitoring suggested that observed exposure was between 60%–90%, there might be no change in media buying. However, if observed exposure was only 50%, substantially different than the expected 75%, the strategy would change. The sample size needed to establish that the achieved exposure was at least 60% would be smaller than the sample size required to establish that it was much closer to 75%; thus, the resources to be allocated to this monitoring question would be reduced.

Research to influence decisions also must happen fast enough to be taken into account. Research about what was failing a year before it was reported may not influence current operational decisions. If results are stale, they may not be given credibility by program managers for current decision-making. Quicker results (even if they carry greater inferential uncertainty) may be more valued than slower results appearing too late to act on.

### Resources Required

I have argued that research planners should focus on crucial links and on research that will influence operational decisions. However, even for those topics, resources will be limited, and further decisions about where to spend resources for data collection (and researchers' time and energy) will require thoughtful allocation. Projects may fail because things that are supposed to happen do not happen as expected. Exposure to media messages is less than expected, parents do not bring their children to the clinic, and few people report changes in their drug use. Research to monitor these events can be low cost and can happen on a continuous basis. If these changes are expected by a project and do not happen, they make it clear that there is a problem that needs to be addressed. If these crucial events are happening as expected, even if their attribution to project efforts is not certain, it may be that there is not so much reason to change operations or to do additional research. Research resources may be better spent on places where there are shortfalls in expected activities or outcomes.

If there are shortfalls and planners are uncertain about how to remedy them, research focused on testing the links in the conceptual or operational model may have a stronger call on available resources. Monitoring research can be straightforward, using repeated surveys or archival information. In contrast, exploring attribution and testing the links may be more costly and demand more elaborate research designs. Even so, designs can be more and less costly, better at making causal claims, and/or better at matching the real context of the project. Choosing which design will provide enough information and whose results will be relevant and actionable at an acceptable cost in money and time will be a valued skill for research planners.

## CONCLUSIONS

Operational research is an essential complement to foundational, formative, and summative evaluation. Programs make mistakes or, for other reasons, do not produce expected effects but may not know it if they do not systematically monitor and test their operational expectations and assumptions. Operational research is not only about data collection but also about knowing what questions are worth addressing and how to incorporate results into program decisions in a timely way. There is likely to be value in specifying program conceptual and logistical models that will lay out possible foci for operational research. These models will generate potential lists of activities to monitor and expectations for paths of effect that can be tested. Choosing among activities to monitor and choosing among expected paths to test will be a major part of the operational evaluator's work. Criteria for choosing among candidate research questions will include expert's judgment about crucial links—from subject matter experts, what underlies a specific type of behavior change and from technical intervention experts, what typically is a challenge for this intervention approach. A second criterion will be the promise that the results of an operational research effort will affect program decisions in a timely way—are program planners ready to act contingent on the results of research? Finally, there will be a complex methodological dance, balancing available resources of funding and researcher time in choosing research strategies and designs that provide sufficient information to influence decisions as the project evolves, maximizing the potential for success.

## Notes

**Peer Reviewed**

**First published date:** December 15, 2023.
